# Observations on Deep Injection of Morphia

**Published:** 1878-09

**Authors:** George E. Jones


### Miscellaneous.

### Some Observations on the Deep Injection of Morphia.

By GEORGE E. JONES, M. D. Read before the Cincinnati Academy of Medicine, July 15, 1878.

In June, 1876, I was called to see Mrs. B., a German woman, aged 37, 5 feet 6 inches in height and weighing 135 lbs. She was the mother of four healthy children.

She stated that she had been under medical treatment several times for "pains in the back, paralysis of the lower limbs and a sickening feeling around the stomach." The above symptoms had been relieved by her former medical attendant. In answer to my questions she described the pain in the lumbar region as one of great intensity, at times extending to her feet; occasionally her limbs would become paralyzed and remain so for two or three hours. She was unable to retain her food for several days at a time, and often two weeks would pass without any action of the bowels, producing, as a general rule, no discomfort.

At the pyloric end of the duodenum there was a tumor about the size of an orange, well defined, indurated and quite painful on pressure. Her urine was small in quantity, of high color with a disagreeable odor. Heat and nitric acid threw down considerable albumen. The microscopic field exhibited tube-casts, blood corpuscles and epithelium in abundance, with some granular matter. This trouble in the course of a month subsided.

The bowels were rather difficult to act upon. The first stool after a brisk cathartic was heavily loaded with pus and blood; her relief now was considerable; of necessity I repeated the cathartic in milder form, which gave same result, but not in quantity; she was now much easier.

About ten days after, a messenger came in rather an excited manner saying that the patient was dying. In a few moments I was at her bedside, and found her suffering most excruciating pain in the lumbar region, passing in waves to her feet, which were quite rapid. Wishing to retain the little control of the stomach, I gave her sulphate of morphia, half a grain, hypodermically. She became easy and slept about three hours. Three days later, under similar circumstances, I repeated the above with the same result.

As the case was one of peculiar interest, I called in a number of my medical friends to see her. Among these were Drs. Carson, Conner, Walton, Cleveland, Drury, Sittel, Underhill and Taylor. Neither of them was willing to express any positive opinion. Drs. Conner and Sittel independently of each other advised that a quantity of water be thrown into the rectum. A tin funnel, large enough to hold a gallon, was made, filled, and from a height of some five feet the water was allowed to flow through a tube into the bowel. She held it several minutes, when it passed off, carrying with it a considerable amount of hard fecal matter that seemed as if it had been packed in the intestines for months; this movement gave her very great relief. At the end of a week I repeated this with good effect. There was no tenderness over the abdomen only in the region of the tumor mentioned.

The tumor began to diminish, but the pains in the lumbar region increased in frequency and intensity, and I had of necessity to increase the amount of morphia, as it was the only drug that would give her any relief, until in the course of a few weeks I reached the amount of 30 grains daily, 15 grains in the morning and 15 grains in the evening; this was continued 21 consecutive days; then I began gradually to lessen the amount to 15 grains daily, finally to one grain a day, giving in twenty months, hypodermically, nearly nine ounces of sulphate of morphia, which required over 2,300 punctures of the needle; these injections were made in the abdomen and outer face of the thighs.

Did the needle enter an abdominal vein? Several times. The first time I became somewhat alarmed, the patient at once threw up her arms, complained of suffocation, giddiness, excessive fatigue, a severe tingling sensation following the course of the circulation. The countenance was at first livid, then flushed; the eyes became unusally brilliant; slight muscular twitchings, profuse sweating with cold extremities, and in a few moments complete relaxation was followed by deep sleep which lasted only four hours, when she awakened, feeling, as she expressed it, "ever so much better."

The same accident occurred three times, the symptoms much milder with the exception of a burning sensation of both eyelids of either eye and both lips, which at one time became painfully intense. The above symptoms were produced by an injection of five grains of sulph. morphia into an abdominal vein.

Being unable to see her for a day or two, I requested my friend, Dr. Geo. E. Walton, who had watched the case with a good deal of interest, to call and give her an injection of two grains, when she put both hands to her head and gave a cry of excruciating agony, a sharp pain darted through her head which lasted ten or fifteen minutes; also complained of an intense itching of nose and lips, finally passing off, leaving no deleterious effects. The same accident occurred to myself, only in a less degree. These injections were also made in the abdomen.

Did the hypodermic injections cause the productions of any abscesses? Never met with any trouble of the kind, which I think was owing to the fact that *I made deep injections with warm concentrated solutions of morphia*, that were freshly prepared every other day. Not only in this case, but in all others, am I in the habit of using the injecting fluid warm, but I am also careful to make the injections deep. Under the circumstances, as I have met with success, I think the above is the proper method.

Are there any ill effects produced by continued use of morphia when hypodermically administered? In conversation with Prof. Bartholow, a few days past, I found him fully able from his great experience to answer this question: "If morphia be gradually diminished in a careful manner very little trouble, if any, need be anticipated; if withdrawn suddenly grave symptoms, even collapse may follow." My own experience, as far as it goes, agrees with the above. I have never met with any ill effect in any case when I have continued the use of morphia hypodermically for any length of time and gradually withdrawing it; especially so in the case mentioned in this paper, when such a quantity had been used.

Neither have I met with the same amount of nausea of the stomach, or trouble with the kidneys as when given internally; at the same time medicine and food can be given with a fair assurance that they will not be interfered with. In conclusion, I would offer the following aphorisms:

Injections directly under the skin, are, as a general rule, painful, and are liable to produce abscess.

Deep injections are not painful and are not so liable to produce abscess.

The injecting fluid must be at least the same temperature as that of the body.

Morphine hypodermically is always more positive in its action, and more equally distributed throughout the system, than when taken in the stomach.

One-eighth of a grain of morphia hypodermically, is, as a general rule, equal to one-half to three-quarters of a grain internally.

